# Induced proximity-based therapeutics for advanced prostate cancer

**DOI:** 10.1038/s44386-026-00062-5

**Published:** 2026-08-14

**Authors:** John Ching, Cindy H. Chau, William D. Figg

**Affiliations:** 1 Clinical Pharmacology Program, Office of the Clinical Director, Center for Cancer Research, National Cancer Institute, National Institutes of Health, Bethesda, MD US; 2 Genitourinary Malignancies Branch, Center for Cancer Research, National Cancer Institute, National Institutes of Health, Bethesda, MD US

**Keywords:** Cancer, Drug discovery, Oncology

## Abstract

Metastatic castration-resistant prostate cancers (mCRPC) remain dependent on androgen receptor (AR) signaling despite resistance to androgen-deprivation and AR-targeted therapies. Induced proximity-based therapeutics employ event-driven pharmacology to target AR, potentially overcoming resistance mechanisms. Herein, we highlight recent advances in induced proximity strategies targeting AR in mCRPC.

## Introduction

Following Charles B. Huggins’s landmark publication on the effects of testosterone suppression for the treatment of advanced prostate cancer^1^, androgen deprivation therapy (ADT) has become a mainstay in prostate cancer management. However, tumors that become unresponsive to such treatment will inevitably progress as castration-resistant prostate cancer (CRPC). While the addition of androgen receptor pathway inhibitors (ARPIs) and taxanes to standard ADT has led to significantly improved outcomes, emerging resistance to these drugs necessitates the discovery of new alternative pathways of treatment. Although there are subsets of metastatic CRPC (mCRPC) lacking in androgen receptor (AR) dependency, many cases of mCRPC still rely on AR signaling but have developed mechanisms that compensate for AR suppression, such as AR amplification, mutations that affect drug binding, and changes in the biosynthesis pathway of androgens^2^. Thus, AR targeting in mCRPC provides both a unique challenge and opportunity: while the targeting of AR signaling and expression remains a highly selective factor for mCRPC, the development of a variety of resistance pathways prevents successful intervention by standard-of-care treatment. As such, additional pathways capable of either suppressing the androgen receptor axis or exploiting its expression in mCRPC despite these resistances are vital to overcoming castration resistance.

**Fig. 1:**
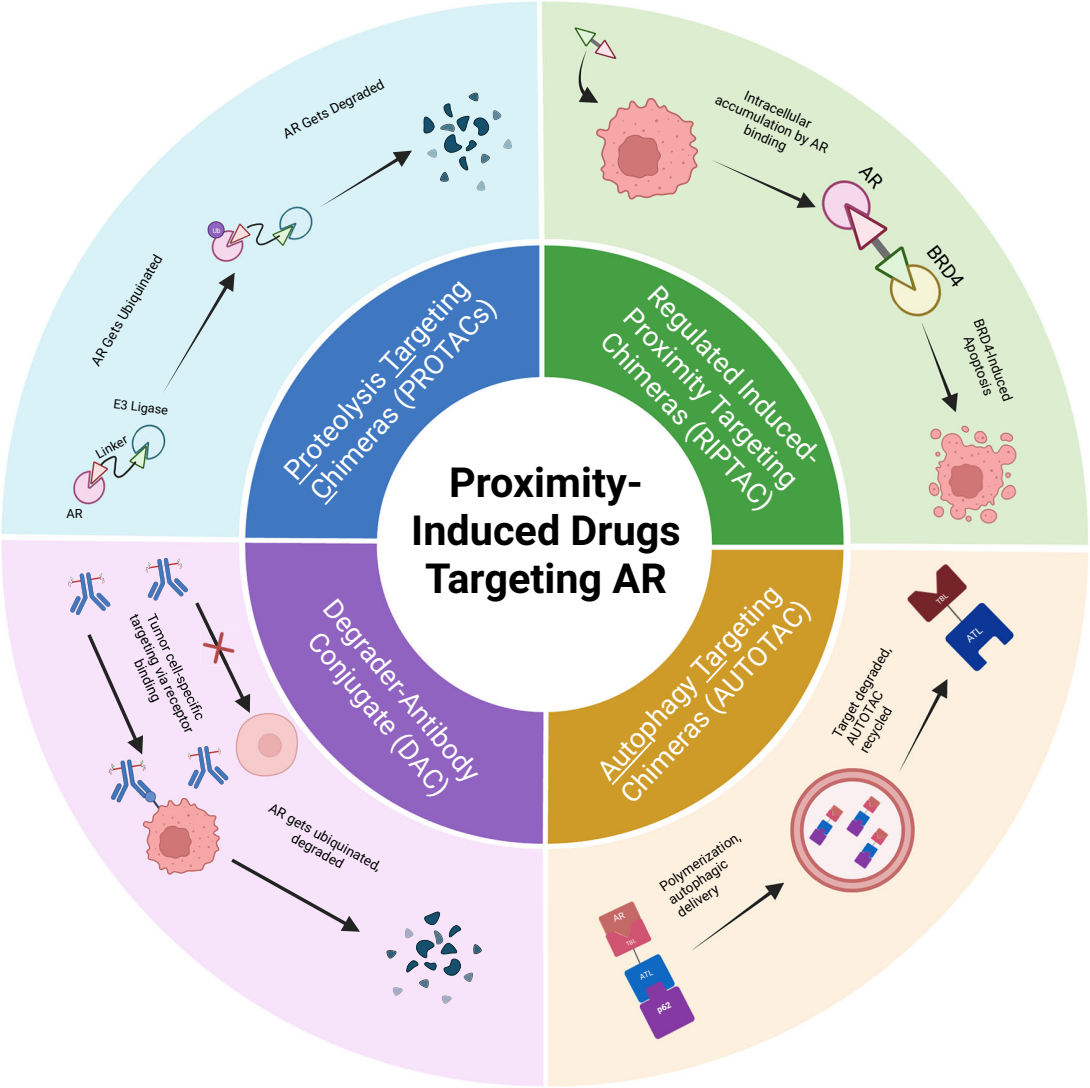
Diagram of various induced proximity-based drugs that target AR. PROTACs suppress AR signaling through degradation of AR molecules using the ubiquitin-proteasome system (UPS)^8^. RIPTACs use AR as a tumor-specific marker, which allows for cell-selective inhibition of BRD4 in mCRPC^39^. The degrader-antibody conjugates (DACs) allow degraders to utilize the same tumor-specific targeting of the RIPTAC modality while maintaining the advantages of protein degradation^48,49^. AUTOphagy TArgeting Chimeras (AUTOTACs) have been described in preclinical testing to degrade both AR and AR-V7, a truncated form of AR that constitutively signals, through the lysosomal pathway^47^. Created in https://BioRender.com.

Induced proximity-based therapeutics are a growing field of drugs that aim to bypass traditional chemotherapeutic resistance. These drugs rely on “event-driven pharmacology”, in which their pharmacological effect is mediated through transient catalytic activity. This results in the interaction of a target and effector molecule, as opposed to “occupancy-based” inhibitors that inhibit their target molecule directly. While diverse in their specific mechanisms, this class of compounds shares the important distinction of bypassing traditional mechanisms of AR resistance through this “event-driven” approach, such as amplifications in AR synthesis and mutations in AR binding ligand affinity^3^. A number of AR-targeting induced proximity-based drugs are currently in development for use in mCRPC, with several mechanisms through which AR expression can be taken advantage of or suppressed for CRPC treatment (Fig. 1). Here, we discuss two subcategories from this drug class, namely PROteolysis-TArgeting Chimeras (PROTACs) and Regulated Induced Proximity TArgeting Chimeras (RIPTACs), and recent advancements in these subgroups for the treatment of CRPC via AR-specific targeting.

### PROTACs

PROTACs were the first of the rationally designed induced proximity-based drugs to be developed, with a proof-of-concept published by the Crews lab^4^ in 2001 first demonstrating the potential of this class of compounds. These heterobifunctional molecules are typically comprised of three distinct subunits, with an E3 ligase targeting domain, a linker, and a protein of interest (POI) warhead that together facilitate the degradation of the POI via the ubiquitination-proteasome system. The binding of the E3 ligase binding domain and the POI binding domain to their respective targets leads to the polyubiquitination of the POI, resulting in the degradation of the target protein by proteasomes^4^. These POIs are oftentimes neosubstrates, as the PROTAC design comes from utilizing the ability of E3 ligases to degrade protein targets, which would, under normal physiological conditions, not serve as standard substrates for E3 ligase activity. Since the initial discovery of PROTACs by the Crews group, a large catalog of PROTACs has been developed for high selectivity against a wide range of targets, from the immunomodulatory molecule IRAK4 to oncogenic variants of proteins such as KRAS G12D^3^. With the recent FDA approval of Arvinias’s ER-degrader Vepdegestrant (ARV-471) for ER+, HER2-, *ESR1-*mutanted advanced breast cancer, the clinical viability of the degrader modality has been shown to be possible, opening the door for potential application in various other disease states, including mCRPC^5^. Aside from circumventing traditional resistance mechanisms, PROTACs are rather potent even at low concentrations and can sustain a long-term suppressive response^6^, providing an attractive modality for durable AR suppression comparable to or superior to standard-of-care direct AR inhibitors such as enzalutamide^7^. Further optimization of the AR-targeting PROTAC model has led to the development of novel modes of AR inhibition that combine the AR-degrading mechanism with more direct antagonistic functions within the same formulation, providing a two-pronged approach that enhances the overall efficacy of the PROTAC design. This model was tested in Nayak et al.’s publication on the discovery and preclinical activity of the dual-action degrader, BMS-986365^8^.

BMS-986365 is a first-in-class heterobifunctional AR-ligand-directed degrader and antagonist. BMS-986365 was identified through empirical screening of compounds able to bind to the E3 ligase adaptor protein cereblon (CRBN) and expresses both competitive antagonistic activity and proximity-induced AR degradation. This unique mechanism was aimed at addressing the challenges associated with coactivator binding surface formation following high-affinity ligand binding to AR. The degrader and competitive inhibition ability of BMS-986365 were individually assessed to determine the primary mechanism behind BMS-986365 efficacy using *FKBP5* mRNA and proliferation activity as metrics. When AR degradation was attenuated following CRBN disruption, BMS-986365 still exhibited similar or superior AR inhibition compared to enzalutamide, suggesting that BMS-986335 inhibits AR independent of degradation. Following this, a patient-derived xenograft (PDX) model obtained from an ADT-administered patient was used to demonstrate the in vivo efficacy of BMS-986365 against enzalutamide for patients developing resistance to standard ADT. Efficacy studies following the progression of PDX tumors 59 days post final dose administration found that BMS-986365-treated tumors maintained some levels of AR protein suppression, whereas enzalutamide-treated tumors expressed significantly upregulated AR protein expression, correlating with significantly improved antitumor activity of BMS-986365 compared to enzalutamide. BMS-986365 also demonstrated preclinical efficacy against several AR mutant forms alongside robust prostate specific antigen (PSA) and radiographic response in a patient who exhibited AR amplification, further suggesting resistance circumvention via the PROTAC mechanism^8^. Overall, these data support BMS-986365’s efficacy in the simultaneous degradation and direct inhibition of AR in ARPI-resistant mCRPC, highlighting its strengths in potentially overcoming historical mechanisms of resistance to ADT and ARPIs^8,9^.

Comparisons between direct AR inhibitors and degraders like BMS-986365 in clinical trials help illustrate the potential of the targeted degradation approach. The phase I trial of BMS-986365 for patients who progressed following ARPI treatments such as enzalutamide, apalutamide, and darolutamide or the CYP17 inhibitor abiraterone reported a >50% decline in PSA (PSA50) of 32%, and median radiographic progression-free survival was 6.3 months. The efficacy of BMS-986365 and other AR-directed degraders against AR amplification and AR mutational variants, some of the major advantages touted by AR degraders over standard-of-care, have proven promising. Although efficacy in the AR amplification subpopulation of mCRPC was not separately listed in the final report, interim analysis revealed clinical efficacy in patients expressing AR amplification status and ligand binding domain mutations. These results are in line with the previously reported preclinical efficacy of BMS-986365 in the VCaP cell line, which exhibits significantly elevated AR gene amplification, and PDX models which demonstrate upregulated AR expression following enzalutamide treatment^8^. Efficacy in patients harboring AR mutational variants, including L702H and/or H875Y, T878A, and W742C, was observed with a PSA50 of 55%. Toxicity was additionally comparable to that observed in standard AR antagonists, with prolonged QTc (47%), bradycardia (34%), nausea (22%), and fatigue (12%) being the most common adverse events reported^10^. The concerns regarding low oral bioavailability and poor pharmacokinetics in PROTAC design was able to be circumvented following more appropriate dosing, since therapeutic doses of BMS-986365 (400, 600, and 900 mg b.i.d.) reached steady-state plasma concentrations^9^. These results suggest an ability to overcome mutational variants, which is one of the pathways responsible for AR antagonist resistance, with sufficient pharmacokinetic profiles to confer clinical benefit and comparable toxicity to standard AR antagonists such as enzalutamide (Table 1)^11^.

**Table 1:** Clinical comparison between enzalutamide, BMS-986365, and HLD-0915

	Enzalutamide	BMS-986365	HLD-0915
Efficacy	Standard of care ARPI	PSA50: 32% in patients previously on ≥1 ARPIs	PSA50: 42% in patients previously on ≥1 ARPIs
Overcoming resistance mechanisms	Ineffective in ARPI-resistant mCRPC	Preclinical efficacy in VCaP cell line (AR amplification) and PDX models with elevated AR protein levels; PSA50: 55% in mCRPC patients with LBD mutations; Does not degrade AR-V7 in vitro; Does not target GR or ERG	PSA50 observed in patients with AR amplification, AR-V7+ and/or LBD mutations; Does not target GR or ERG
Toxicity	Fatigue (36%), back pain (27%), hot flushes (18%), cardiac events (10%)	Prolonged QTc (47%), bradycardia (34%), fatigue (22%), nausea (12%)	Nausea (20%), anemia (10%), fatigue (10%)
Pharmacokinetics	T_max_ reached in 0.4–4 h; Half life: 5.8 ± 1.6 days; Steady-state plasma concentrations achieved following 28 days of oral administration	T_max_ reached in 3–8 h; Half life: 20-28 h; Steady-state plasma concentrations achieved following repeated oral administration (400, 600, & 900 mg twice a day)	T_max_ reached in 4–6 h; Half life: Not reported; Steady-state plasma state concentrations following repeated oral administration (25 & 50 mg once a day)
References	^11^	^8,9^	^39^

*AR* androgen receptor; *AR-V7* androgen receptor splice variant 7; *ARPI* androgen receptor pathway inhibitor; *ERG* ETS-related gene; *GR* glucocorticoid receptor; *LBD* ligand binding domain; *mCRPC* metastatic castration-resistant prostate cancer; *PDX* patient-derived xenograft; *PSA* prostate-specific antigen; *PSA50* Percentage of patients who have a ≥50% reduction from baseline in serum PSA; *QTc* corrected QT interval.

However, PROTAC capabilities against several AR resistance mechanisms either remain understudied or have proven difficult to overcome with current approaches. A significant resistance mechanism in progressive CRPC comes from the recruitment of coactivators, which modulate the AR cistrome to promote treatment resistance^12^. Although successful degradation of AR following PROTAC binding would theoretically be capable of preventing coactivator function due to preventing AR transcriptional activity, the responses in AR coactivator function following PROTAC degradation are currently understudied. As PROTAC remain highly specific to their respective degradation target, bypassing of the AR signaling pathway through the glucocorticoid receptor (GR) and ERG are also not covered by present PROTAC technology, although the specifics of these interactions have not been elucidated at present^13,14^. Additionally, BMS-986365 was noted in preclinical testing to be less efficacious in degrading the AR-V7 mutant, a variant of AR that lacks the ligand-binding domain (LBD), and this particular pitfall is one that both BMS-986365 and other current LBD-based AR degraders have not been able to address. Immunohistochemistry staining of castration-resistant prostate cancer tissue samples following ADT demonstrated that 75% of CRPC were AR-V7+ compared to <1% of castration sensitive prostate cancer tissue samples^15^. Less dramatic but nonetheless concerning, qPCR analysis of circulating tumor cells (CTCs) in 31 patients treated with enzalutamide and 31 patients treated with abiraterone found that 39% and 19% of enzalutamide- or abiraterone-treated patients expressed detectable levels of AR-V7, respectively, with AR-V7 status significantly correlated with worse PSA response rates and survival outcomes^16^. AR-V7 frequency has also been observed to increase following subsequent lines of therapy, with immunofluorescent staining of CTC samples finding that frequency of AR-V7 increased from 3% to 31% from first-line treatment to third- or greater line treatment, respectively^17^. This indicates a potentially higher level of resistance to LBD-focused degraders in the mCRPC patient population. Thus, the question remains whether degradation limited to AR mutants containing the LBD will be sufficient to demonstrate clinical efficacy in mCRPC patients. Additionally, comparisons between clinical trial results for mCRPC of other AR degraders with BMS-986365 highlight a notable difference in the frequency of cardiovascular events (CVEs), with asymptomatic prolonged QTc (47%) and bradycardia (34%) being frequently cited treatment-related adverse events (TRAEs) for BMS-986365-treated patients^9,18–20^. ARPIs have been associated with higher CVE rates, and perhaps the additional direct antagonist activity of BMS-986365 relative to other degraders might in part contribute to this observed phenomenon^21^. However, it remains challenging to delineate a direct association of CVEs to BMS-986365 against a background of previous or ongoing ADT and ARPIs regimens, considering the known class effects of ADT and ARPIs on CVEs^21^. Within the phase I/II testing, all cases of grade ≥3 of prolonged QTc were found to be reversible to either baseline or Grade ≤2 after dose reduction, which suggests these complications will not pose as a dose-limiting factor for BMS-986365 usage^9^. Nevertheless, the degrader’s overall efficacy and manageable safety profile observed in the phase I trial have led to the BMS-986365 drug moving into a Phase III trial (NCT06764485) that is currently open to accrual. While it remains to be seen how BMS-986335 performs in the clinic, it is currently poised to be furthest along in the clinic amongst the AR degraders in use for metastatic CRPC (Table 2)^9^.

**Table 2:** Current AR-targeted induced proximity-based drugs in clinical development

AR-targeted therapy	Combination (if applicable)	Clinical population	Target	Clinical trial phase	NCT Number	Company
BMS-986365/CC-94676	Degralix	High-risk localized PC	AR LBD/antagonist	Phase 2	NCT07335796	Bristol Myers Squibb
	N/A	mCRPC		Phase 3	NCT06764485	
JSB462 (ARV766/Luxdegalutamide)	Abiraterone (subarm)	mCRPC	AR LBD	Phase 1/2	NCT05067140	Novartis/Arvinas
	N/A	mCRPC		Phase 1	NCT07174063	
	Abiraterone	Metastatic Hormone-sensitive PC		Phase 2	NCT06991556	
	Tulmimetostat	mCRPC		Phase 1/2	NCT07206056	
	Lutetium (177Lu) Vipivotide Tetraxetan	mCRPC		Phase 2	NCT07047118	
HP518	N/A	mCRPC	AR LBD	Phase 1/2	NCT06155084	Hinova Pharmaceuticals
AZD9750	Saruparib (subarm)	mCRPC	AR LBD	Phase 1/2	NCT07336446	AstraZeneca
HRS-5041	Abiraterone & Prednisone; Docetaxel; HRS-1167; SHR2554	Advanced PC	AR LBD	Phase 1/2	NCT06568094	Jiangsu HengRui
	HRS-2189	mCRPC		Phase 2	NCT06738745	
TQB3201	N/A	Advanced PC	AR LBD	Phase 1/2	NCT07172126	Chia Tai Tianqing Pharmaceutical Group
GSK5471713	N/A	mCRPC	AR LBD	Phase 1/2	NCT07332455	GlaxoSmithKline
RO7656594 (GDC-2992/RG6537)	N/A	Advanced PC or mCRPC	AR LBD	Phase 1	NCT05800665	Genentech/ Jemincare
QLH12016	Abiraterone & Enzalutamide	Advanced PC	AR LBD	Phase 1/2	NCT07104110	Qilu Pharmaceutical Co.
	QLC5508; Abiraterone & Enzalutamide	Advanced PC		Phase 1/2	NCT07198633	
HLD-0915	N/A	mCRPC	AR RIPTAC	Phase 1/2	NCT06800313	Halda Therapeutics

*AR* androgen receptor; *LBD* ligand binding domain; *mCRPC* metastatic castration-resistant prostate cancer; *PC* prostate cancer; *RIPTAC* regulated induced proximity targeting chimera.

### Current challenges with PROTACs

There remain existing challenges that have prevented more direct bench-to-bedside translation of the PROTAC modality. PROTACs possess a number of characteristics that negatively impact their oral bioavailability and cell permeability, such as high molecular weight, large numbers of hydrogen donor groups, large polar surface area, and balancing high lipophilicity and aqueous solubility^22,23^. While current-generation AR degraders (such as BMS-986365, ARV-766, and AZD9750) have been optimized to improve the structures of the compounds to improve their pharmacokinetic properties, many of these clinical candidates were improved through empirical testing, presenting a gap in the current PROTAC development landscape for more efficient methods of drug discovery^24^. Various technologies are currently under investigation to enhance PROTAC design and optimization, with particular emphasis on large-scale dataset approaches such as AI-driven predictive modeling of PROTAC pharmacokinetic profiles and PROTAC:protein interactions, the large array of potential E3 ligase binders, linker, and target protein ligands that can comprise the structure of any given PROTAC have proven to be a challenge to effectively predict^25,26^. On the other hand, several approaches are being tested to improve the pharmacokinetic properties of PROTACs to improve performance in the clinical setting. Optimizations in linker technology to facilitate favorable pharmacokinetic properties in PROTACs, prodrug formulations, and nanoparticle technology delivery systems are all strategies currently being explored and have been extensively reviewed elsewhere^27–30^.

In terms of molecular roadblocks, acquired degrader resistance is an emerging issue that will likely plague degrader development over the coming years. Although PROTACs and other degrader drugs can overcome resistances developed in the protein of interest, acquired resistances to PROTACs via alterations in E3 ligase expression or structure can prevent effective degradation. For example, exposure of the ovarian cancer cell line OVCAR8 to increasing concentrations of CRBN and Von Hippel Lindau (VHL)-based BET protein degraders over the course of four months led to loss of the gene encoding CRBN and genomic alterations to the CUL2 chromosomal locus, which is an essential scaffolding protein in the VHL-E3 ligase complex. These genomic alterations resulted in PROTAC IC50 values 40 times greater than that of the parental cell lines, although intriguingly the prostate cancer cell line tested in this report (LNCaP) did not generate a stable resistant cell line, in part postulated to be due to prostate cancer’s greater reliance on BET protein function^31^. The reliance on these degraders for a specific ligand for PROTAC function to be facilitated also demonstrates a resistance mechanism that has not yet been overcome in current PROTAC design, as can be seen by current AR degraders lacking efficacy in AR-V7 degradation, which lacks the ligand-binding domain used by most AR PROTACs for tertiary complex formation. PROTACs as a class also suffer from several design challenges that limit direct preclinical agent development. The complexity of the PROTAC structure requires more advanced synthesis technologies, although scaling of the manufacturing operations designed to develop these PROTACs somewhat alleviates these issues^32^. High concentrations of PROTAC molecules can result in what is known as the “hook effect”, in which high concentrations of PROTAC molecules lead to saturation of E3 ligase:PROTAC or POI:PROTAC binary complex that cannot facilitate degradation, resulting in lower efficacy at higher doses despite the larger therapeutic window afforded by most PROTACs^33^. Finally, PROTACs typically do not include a mechanism by which tumor cell-specific uptake can be achieved. While current-generation AR degraders display toxicity comparable to that of current-generation ARPIs, suggesting limited off-target activity, targeting of more globally essential proteins such as bromodomain and extraterminal domain (BET) proteins may result in significant toxicity^9,11,20^. For example, the BET family of proteins is a group of transcriptional regulators of great interest due to their demonstrated regulatory role in a variety of cancers; however, previous pan-BETis have been limited by high frequencies of dose-limiting toxicities (DLTs) such as thrombocytopenia, nausea, and fatigue^34^. Targeting a specific BET family protein could reduce DLT frequency, opening these proteins for targeting. From these, BRD4 stands out due to its transcription interactions with AR and the apoptotic and antiproliferative effects of BRD4 inhibition in prostate cancer cell lines^35–37^. Alternative strategies that can mitigate these issue of toxicity may provide an avenue through which these proteins can be unlocked for targeted therapy.

### RIPTACs

Compared to PROTACs, Regulated Induced Proximity TArgeting Chimeras (RIPTACs) rely on tertiary structure stabilization as opposed to degradation for their pharmacological function. RIPTACs, similar to PROTACs, are heterobifunctional molecules that utilize binding to a tumor protein (TP) that causes intracellular accumulation and inhibition of an essential protein (EP) through cooperative binding that results in cellular apoptosis. Due to the unique molecular architecture of mCRPC, RIPTACs using AR as the tumor-specific protein are uniquely positioned to inhibit targets with high levels of tumor cell specificity. RIPTACs, while maintaining many of the catalytic advantages of PROTACs, present several major advantages as a standalone agent. Most notably, their status as small molecules makes them more attractive as potential drug candidates, and their added specificity lowers the risk for non-tumor cell toxicity, which could help to push these proximity-based drugs into the market for prostate cancer^38^.

Preliminary clinical data on the Phase I trial for the HLD-0915 RIPTAC targeting BRD4 and AR in metastatic CRPC were reported. Patients with mCRPC were given oral administration daily on a 21-day cycle and were followed for treatment-related adverse events (TRAEs). Out of 31 patients treated, 13 (42%) achieved PSA50, and 7 (23%) achieved a >90% decline in PSA (PSA90). Grade <3 TRAEs included nausea (20%), anemia (10%), and fatigue (10%), with Grade ≥3 TRAEs including lymphocyte decreases (3%) and hypertriglyceridemia (3%). One case of Grade ≥3 increases in ALT, AST, and total bilirubin was noted but not attributed to drug toxicity. Steady-state plasma concentrations of HLD-0915 were also achieved following repeated administration of therapeutically active doses. Most importantly, HLD-0915 demonstrated efficacy irrespective of AR amplification, LBD mutations, and AR-V7 variants, highlighting HLD-0915’s ability to overcome AR resistance^39^.

RIPTACs, much like PROTACs, possess certain characteristics that hinder clinical translation. For one, due to the large molecular weight of RIPTACs, RIPTACs maintain many of the same pharmacokinetic issues as PROTACs, such as low oral bioavailability and cell permeability. There are also restrictive design considerations to make, as RIPTACs require the formation of stable tertiary structures to facilitate EP inhibition, which places heavy requirements for the rational design of RIPTACs, which is further limited by the small number of currently identified list of tumor proteins that can viably be used for RIPTAC development. However, the high specificity of RIPTACs to their respective targets and the lack of requirement for the tumor protein to be a primary driver of disease are major advantages that are difficult to replicate in currently used PROTAC technology, and the progression of HLD-0915 into a phase II trial (NCT06800313) are positive signs that RIPTACs will have a path to becoming established in the clinical setting (Table 3)^40^.

**Table 3:** Comparisons of PROTACs and RIPTACs for clinical development

	PROTACs	RIPTACs
Mechanism of action	Tertiary complex formation between E3 ligase and POI; POI proteasome-based degradation	Tertiary complex stabilization through cooperative binding; EP inhibition
Degradation of target?	✓	X
Tumor cell specificity?	X	✓
Pharmacokinetics	Low oral bioavailability, cell permeability; Linker vulnerable to metabolic breakdown; Half-life	Low oral bioavailability, cell permeability; Linker vulnerable to metabolic breakdown; Half-life
Design constraints	Limited repertoire of E3 ligases currently available; Exhibits the “hook effect”	Limited number of TPs identified; Requires stable tertiary structure formation between EP and TP

*EP* essential protein; *POI* protein of interest; *PROTAC* proteolysis targeting chimera; *RIPTAC* regulated induced proximity targeting chimera; *TP* target protein.

### Future directions—DACs and AUTOTACs

Alongside the promise of these upcoming technologies, PROTACs have been making strides in improving their mechanistic specificity in combination with other proven technologies. In particular, the development of degrader-antibody conjugates (DACs) is beginning to pave the way for more expansive degrader entry in the clinic. Antibody drug conjugates (ADCs), from which this concept is derived from, are powerful modalities by which highly cytotoxic therapeutics can be delivered to tumor cells, limiting off-target toxicity, and use of these ADCs in CRPC has been extensively studied due in part to the presence of several well-defined surface markers which make CRPC a prime target for ADC utilization, such as prostate specific membrane antigen (PSMA) and B7H3^41^. However, these compounds have their own unique issues that limit their use. Specifically, the rather stringent requirements for suitable ADC payloads have significantly limited the roster of potential therapeutics viable for use in the conjugate formulation, such as potency at low concentrations, sufficient stability following linker decoupling, and ability to circumvent traditional resistance mechanisms^42^. PROTACs fit many of the requirements these therapeutics must fulfill, and thus DACs have the potential to combine the best attributes of antibody-conjugate and degrader technologies. For instance, preclinical optimization of a STEAP1-targeting antibody conjugated to a BRD4 degrader demonstrated antiproliferative and in vivo antitumor activity in PC3 cell lines, which serves as a proof-of-concept for the feasibility of an antibody-BRD4 degrader conjugate in mCRPC that maintains tumor-targeted specificity^43^. However, further optimization of PROTACs will likely be necessary to ensure that these compounds are properly efficacious as antibody-drug conjugate payloads. PROTACs, due to their large size, do not demonstrate the “Bystander effect”, whereby a certain percentage of chemotherapeutics escape apoptotic tumor cells to be taken up by neighboring tumor cells. The molecular interactions of antibodies with heterogeneous tumor cell populations mean that only a certain percentage of said tumor cells will be sensitive to direct ADC-receptor binding, and as such, localized tumor cell death is often required for the full effect of ADCs to take place^44^. Incorporation of extracellular vesicle trafficking molecules such as a shortened CD9 molecule into the design of the mechanistically similar constructs of bioPROTACs, which instead consist of an antibody fragment or nanobody specific to the protein of interest fused directly to an E3 ligase adaptor protein, have been shown to give bioPROTACs an ability to express the “Bystander effect” through internalization of bioPROTAC-containing extracellular vesicles, which helps to demonstrate a pathway through which DACs may prove successful in a clinical setting^45^. To date, only one DAC is currently progressing through clinical trials, namely ORM-6151 (clinicaltrials.gov, NCT06419634); however, considering their potential, it will be unsurprising to see more of these conjugates appear in clinical trials in the coming years.

On the horizon, AUTOphagy TArgeting Chimera (AUTOTAC) preclinical agents have been reported in recent literature to degrade AR and AR-V7. These compounds utilize the autophagy system to initiate targeted degradation, forming a complex with the protein of interest and the autophagy-associated protein p62, resulting in p62 polymerization and subsequent autophagosome-based degradation. The particular AUTOTAC platform described here demonstrated AR-V7-degrading activity through heterodimerization of AR-FL and various clinically relevant point mutations, such as L702H, H874Y, F877L, and T878A, and was capable of inhibiting 22Rv1 growth both in vivo and in vitro, which, considering the 22Rv1 cell line’s reliance on AR-V7 activity, suggests a functional response to AR-V7 heterodimer degradation. Particularly in the realm of AR-V7 targeting, AUTOTACs hold the distinct advantage of independence from the tertiary complex formation necessary for the functioning of PROTACs. While the lowered reliance of PROTAC function on binding affinity allows them to degrade AR despite LBD-based point mutations, mutational variants such as AR-V7, which significantly alter tertiary structure, are significant resistance mechanisms that current PROTACs cannot overcome. Although AUTOTACs similarly have difficulties with degrading AR-V7 directly due to current-generation AUTOTACs utilizing LBD-based ligands, the ability of these molecules to target larger protein complexes, such as those formed via heterodimers with AR-V7, suggests a role for the AUTOTAC platform to circumvent the development of certain splice variants in mCRPC progression. At the moment, no AUTOTACs have entered clinical trials for mCRPC, although the promise of the technology may see these compounds arrive in short order^46,47^.

Together, these compounds have helped highlight the benefits of induced proximity-based drugs through the enhancement of efficacy and safety of established treatment mechanisms or paving the way for new mechanisms of action to be developed. It is anticipated that these drugs will validate the effectiveness of the induced proximity-based drug class in the treatment of mCRPC.

## Data Availability

No datasets were generated or analysed during the current study.
